# Rictor Ameliorates Acute Antibody‐Mediated Rejection Following Kidney Transplantation by Suppressing Macrophage M1 Polarization Through p65‐NLRP3 Axis

**DOI:** 10.1002/advs.202417119

**Published:** 2025-06-20

**Authors:** Bin Ni, Chengcheng Yang, Junqi Zhang, Zhou Hang, Ming Zheng, Dengyuan Feng, Qinghuan Shen, Jinxu Miao, Xulin Sun, Li Sun, Baixin Shen, Min Gu, Zijie Wang

**Affiliations:** ^1^ Department of Urology the Second Affiliated Hospital of Nanjing Medical University Nanjing 210011 China; ^2^ Department of Urology the First Affiliated Hospital of Nanjing Medical University Nanjing 210029 China

**Keywords:** antibody‐mediated rejection, kidney transplantation, macrophage, NLRP3, Rictor

## Abstract

Antibody‐mediated rejection (ABMR) represents the leading cause of kidney allograft failure over a long term after transplantation. Early infiltration of macrophages predicts the adverse outcome of grafts, yet the underlying mechanisms remain to be elucidated. Significant infiltration of M1 macrophages and upregulation of Rictor in macrophages are observed in ABMR allografts. Deficiency of Rictor in macrophages exacerbates histological injury and shortens the survival of ABMR allografts by promoting macrophage M1 polarization. Additionally, loss of Rictor in primary bone marrow‐derived macrophages facilitates NLRP3 inflammasome activation through activating NF‐κB. Mechanistically, Rictor upregulates E3 ubiquitin ligase SOCS1 to enhance K48‐linked ubiquitination of p65, thereby suppressing macrophage M1 polarization. Taken together, Rictor ameliorates acute ABMR following kidney transplantation by suppressing macrophage M1 polarization through the p65‐NLRP3 axis and may serve as a therapeutic target for ABMR.

## Graphical abstract

This study elucidates the critical role of Rictor in macrophage activation in acute antibody‐mediated rejection (ABMR). Rictor increases K48‐linked ubiquitination of p65 by upregulating E3 ubiquitin ligase SOCS1, inhibiting transcriptional levels of NLRP3 and inflammasome activation. The finding that macrophage Rictor prolongs the survival of allografts provides potential therapeutic for ABMR following kidney transplantation.

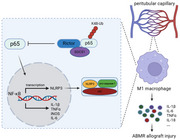

## 1 Introduction

Kidney transplantation effectively prolongs the survival and improves the quality of life of patients with end‐stage kidney disease.^[^ ^1^ ^]^ However, the incidence of antibody‐mediated rejection (ABMR), considered as the most common cause of immune‐related graft loss, rises gradually with time after kidney transplantation.^[^ ^2^, ^3^ ^]^

ABMR is currently believed to develop as B cells and plasma cells are activated to overproduce donor‐specific antibodies (DSAs).^[^ ^4^ ^]^ DSAs bind to endothelial cells within the transplanted kidney to trigger complement‐dependent or ‐independent mechanisms, and recruit various immune cells such as NK cells, neutrophils, platelets, and macrophages, thereby resulting in a series of pathological injuries, including peritubular capillaritis, glomerulitis, and thrombotic microangiopathy.^[^ ^5^ ^]^ As these injuries deteriorate into serious conditions, such as transplant glomerulopathy, arterial intimal fibrosis, and interstitial fibrosis/tubular atrophy, the risk of graft loss increases substantially.^[^ ^1^, ^4^ ^]^ Although specific therapies, such as antibody removal, complement inhibition, B cell depletion, and plasma cell inhibition, have been shown to deal with short‐term outcomes of ABMR, no regimens have demonstrated satisfied results.^[^ ^6^, ^7^, ^8^ ^]^

The intensity of monocyte/macrophage infiltration within the graft during ABMR correlates with the degree of injury and graft outcomes.^[^ ^9^, ^10^ ^]^ Monocytes/macrophages possess the ability to alter their phenotypes and functions in response to local microenvironmental changes,^[^ ^11^ ^]^ termed macrophage polarization. Depending on surface receptors and secretion profiles, macrophages polarize into classically activated M1 and alternatively activated M2 subtypes.^[^ ^12^ ^]^ Having infiltrated into ABMR grafts, monocytes/macrophages may express pro‐inflammatory functions, and synergize with DSAs to aggravate antibody‐mediated graft injury and rejection reactions. However, the specific molecular mechanisms underlying macrophage activation in allografts remain unclear.

Mammalian target of rapamycin (mTOR) is an atypical serine/threonine protein kinase that exists in two complexes within cells. mTORC1, sensitive to rapamycin, consists of mTOR, Raptor, mLST8, PRAS40, and Deptor, while mTORC2, insensitive to rapamycin, consists of mTOR, Rictor, mLST8, Deptor, mSIN1, and Protor1/2.^[^ ^13^, ^14^, ^15^ ^]^ Rictor works considerably changeable in different cells. It was reported that Rictor in acute kidney injury (AKI) has significant effects on dendritic cell phenotype, function, and response to exogenous stimuli. AKI mice with dendritic cell‐specific deletion of Rictor showed higher levels of blood creatinine, blood urea nitrogen, and more severe histological damage, suggesting that Rictor might exacerbate AKI by promoting immune cell infiltration and pro‐inflammatory cytokine production.^[^ ^16^ ^]^ However, specific ablation of Rictor in fibroblasts protects against tubular cell death and dictates the outcome of AKI through stimulating peroxisome proliferator‐activated receptor gamma (PPARγ) and hepatocyte growth factor expression.^[^ ^17^ ^]^ Additionally, specific deletion of Rictor in the myeloid lineage enhanced lipopolysaccharide (LPS)‐induced inflammatory responses.^[^ ^18^ ^]^ However, no studies have clarified the role and specific molecular mechanisms of the Rictor/mTORC2 signaling pathway in regulating macrophage activation, as well as the progression of acute ABMR following kidney transplantation.

In this study, we reported that the expression of Rictor elevated in the macrophages from ABMR kidney allografts, and that the deficiency of Rictor in macrophages exacerbated the histological injury and shortened the survival of ABMR allografts by upregulating M1 macrophages. Thus, we hypothesize that Rictor may ameliorate ABMR allografts by suppressing macrophage M1 polarization through p65‐NLRP3 axis. Collectively, the findings reveal the transcription regulation mechanism of NLRP3 and the vital roles of Rictor/p65/NLRP3 axis in macrophages activation during ABMR, underscoring macrophage Rictor as a therapeutic target for ABMR.

## 2 Results

### 2.1 The Infiltration of M1 Macrophages is Evident in ABMR Allografts

**Figure 1:**
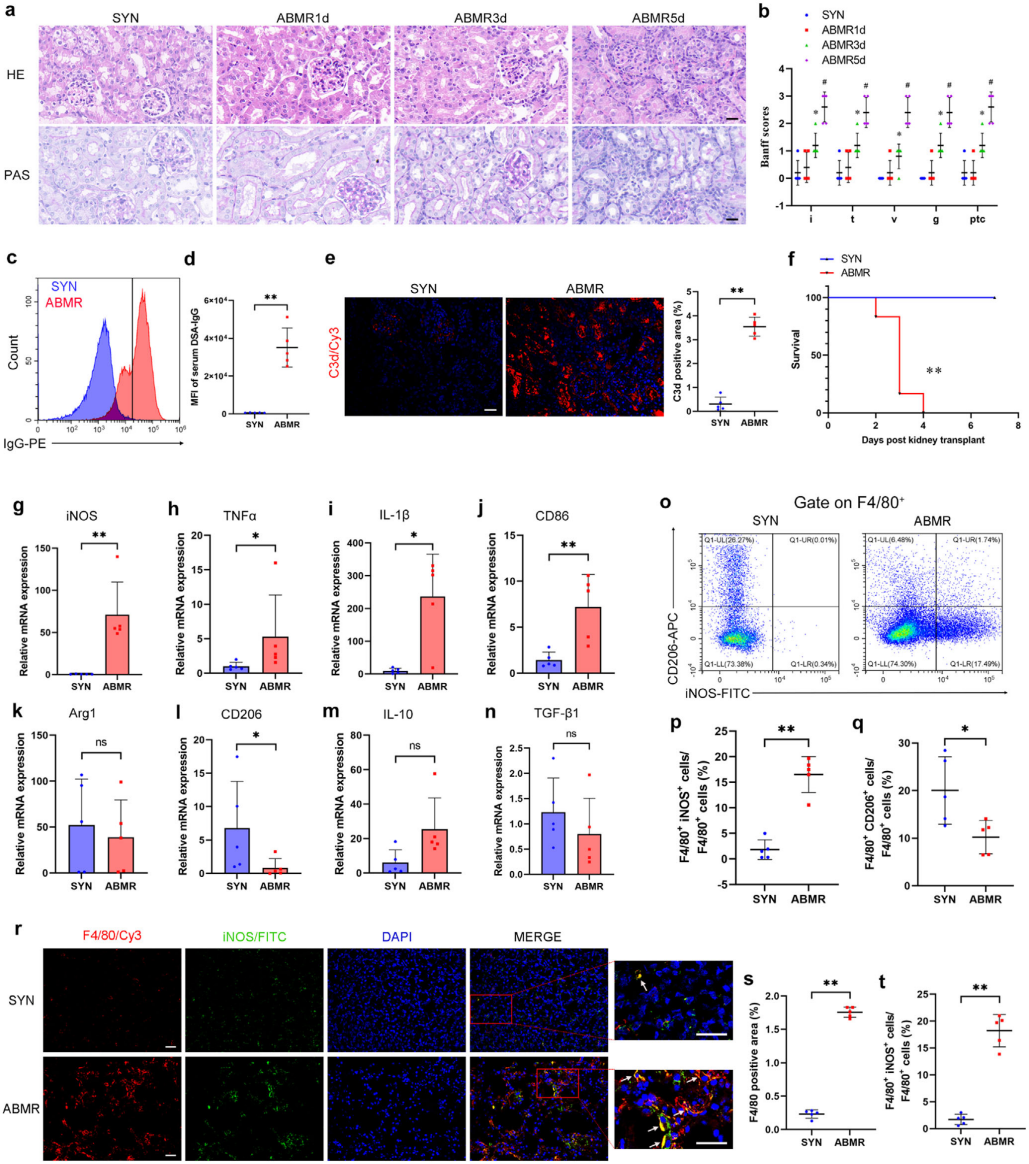
The infiltration of M1 macrophages is evident in ABMR allografts. At day 5 after skin transplant, kidney transplantation was carried out on C57BL/6 mice, who received a kidney from a C57BL/6 donor as a syngeneic (SYN) graft or from a BALB/c donor as an allograft, and the mice were sacrificed at 1, 3, and 5 days post‐operation. a) Histologic images of graft sections stained with hematoxylin and eosin (H&E) and periodic acid Schiff (PAS). Original magnification × 400. Bar = 20 µm. b) Banff scores of interstitis, tubulitis, vasculitis, glomerulitis, peritubular capillaritis in grafts, * Versus SYN group, *p* < 0.05, *n* = 5, # Versus SYN group, *p* < 0.05, *n* = 5. c,d) Flow cytometry showing the median fluorescence intensity (MFI) of serum donor‐specific antibodies (DSA)‐IgG and quantitative analysis in the indicated groups (*n* = 5). e) Representative immunofluorescence (IF) staining and quantitative analysis for C3d in grafts. Original magnification ×400, Bar = 20 µm, *n* = 5. f) Survival analysis of kidney grafts among groups as indicated, Hazard Ratio (HR) = 27.60, 95% CI [4.14, 184.10], *n* = 6. g–n) Quantitative real‐time polymerase chain reaction (qRT‐PCR) analysis showing the mRNA levels of *iNOS* (g), *TNFα* (h), *IL‐1β* (i), *CD86* (j), *Arg1* (k), *CD206* (l), *IL‐10* (m) and *TGF‐β1* (n) in grafts (*n* = 5). o–q) Flow cytometry showing the proportions of F4/80^+^ iNOS^+^ M1 macrophages (p) and F4/80^+^ CD206^+^ M2 macrophages (q) in grafts (*n* = 5). r–t) Representative IF staining and quantitative analysis for F4/80 and iNOS in grafts. White arrows indicate co‐staining‐positive cells. Original magnification × 400, Bar = 20 µm, *n* = 5. Data in (b,d,e,g–n,p,q,s,t) were analyzed using unpaired Student's *t*‐test. Data in f were analyzed using Gehan–Breslow–Wilcoxon test. * p < 0.05; ** *p* < 0.01. Data are presented as mean ± SD.

To explore the role and mechanism of macrophage polarization in ABMR following kidney transplantation, an acute ABMR mouse model was established and validated. The histological injury in the kidney grafts of ABMR mice worsened gradually with time after transplantation (**Figure** **1**a). ABMR mice showed typical peritubular capillaritis, hemorrhage and edema, tubular necrosis, and extensive infiltration of mononuclear cells (Figure 1b). At post‐operation 5 days, ABMR mice with typical histological changes in allografts were harvested for further investigation. Compared with SYN mice, the median fluorescence intensity of serum DSA‐IgG (Figure 1c,d) and C3d (Figure 1e) depositions increased significantly in ABMR mice. Survival analysis showed a significant reduction in ABMR allografts (Figure 1f). The mRNA levels of M1 and M2 polarization markers in kidney grafts were detected. Compared with those in the SYN group, the mRNA levels of *iNOS*, *TNFα*, *IL‐1β*, and *CD86*, polarization markers of M1 macrophages, were significantly increased in ABMR allografts (Figure 1g–j), while the mRNA level of *CD206*, a marker of M2 macrophages, was decreased. In addition, the mRNA levels of *Arg1*, *TGF‐β1*, and *IL‐10* showed no difference (Figure 1k–n). Moreover, these alterations were confirmed by flow cytometry. The proportion of F4/80^+^ iNOS^+^ M1 macrophages increased significantly in the ABMR group, while the proportion of F4/80^+^ CD206^+^ M2 macrophages decreased (Figure 1o–q), which is consistent to our previous study based on rats.^[^ ^19^ ^]^ Additionally, the infiltration of F4/80^+^ macrophage and iNOS^+^ M1 macrophages increased significantly in ABMR allografts (Figure 1r–t). Kidney graft biopsies from stable and ABMR patients were obtained (Figure S1a, Supporting Information). Clinical characteristics are presented in **Table** **1**. Consistently, the infiltration of CD68^+^ macrophage and the proportion of iNOS^+^ M1 macrophages increased significantly in ABMR graft biopsies, compared to those in STA graft biopsies. The above data suggest that M1 macrophages may play a critical role in ABMR.

**Table 1:** Characteristics of patients with stable and ABMR.

		STA (*n* = 9)	ABMR (*n* = 9)	*p*
Sex	Male	5	7	0.620^a)^
	Female	4	2	
Age, yr		35.00 ± 6.15	39.44 ± 13.78	0.417^b)^
Scr, µmol L^−1^		83.46 ± 4.19	273.74 ± 143.08	0.002^b)^
BUN, mmol L^−1^		7.83 ± 0.58	25.37 ± 9.73	<0.001^b)^
DSA	Positive	0	7	0.002^a)^
	Negative	9	2	
HLA	< 50%	6	8	0.577^a)^
	≥ 50%	3	1	
Duration after kidney transplant, yr		0.53 ± 0.28	1.87 ± 2.37	0.134^b)^
DGF	Positive	1	4	0.294^a)^
	Negative	8	5	
Immunosuppressive strategy	CNI+MMF+GC	6	5	0.809^a)^
	CNI+MMF+GC+RAPA	2	2	
	CNI+MMF+GC+IGT	1	2	

Scr, serum creatinine; BUN, blood urea nitrogen; DSA, donor‐specific antibodies; HLA, human leukocyte antigen; DGF, delayed graft function; CNI, calcineurin inhibitor; MMF, mycophenolate mofetil; GC, glucocorticoids; IGT, iguratimod.

^a)^ Fisher exact test;

^b)^ Unpaired student's *t*‐test. Values are *n* (%) or mean ± SD.

### 2.2 Rictor is Upregulated in Macrophages from Mice and Human Kidney Grafts

**Figure 2:**
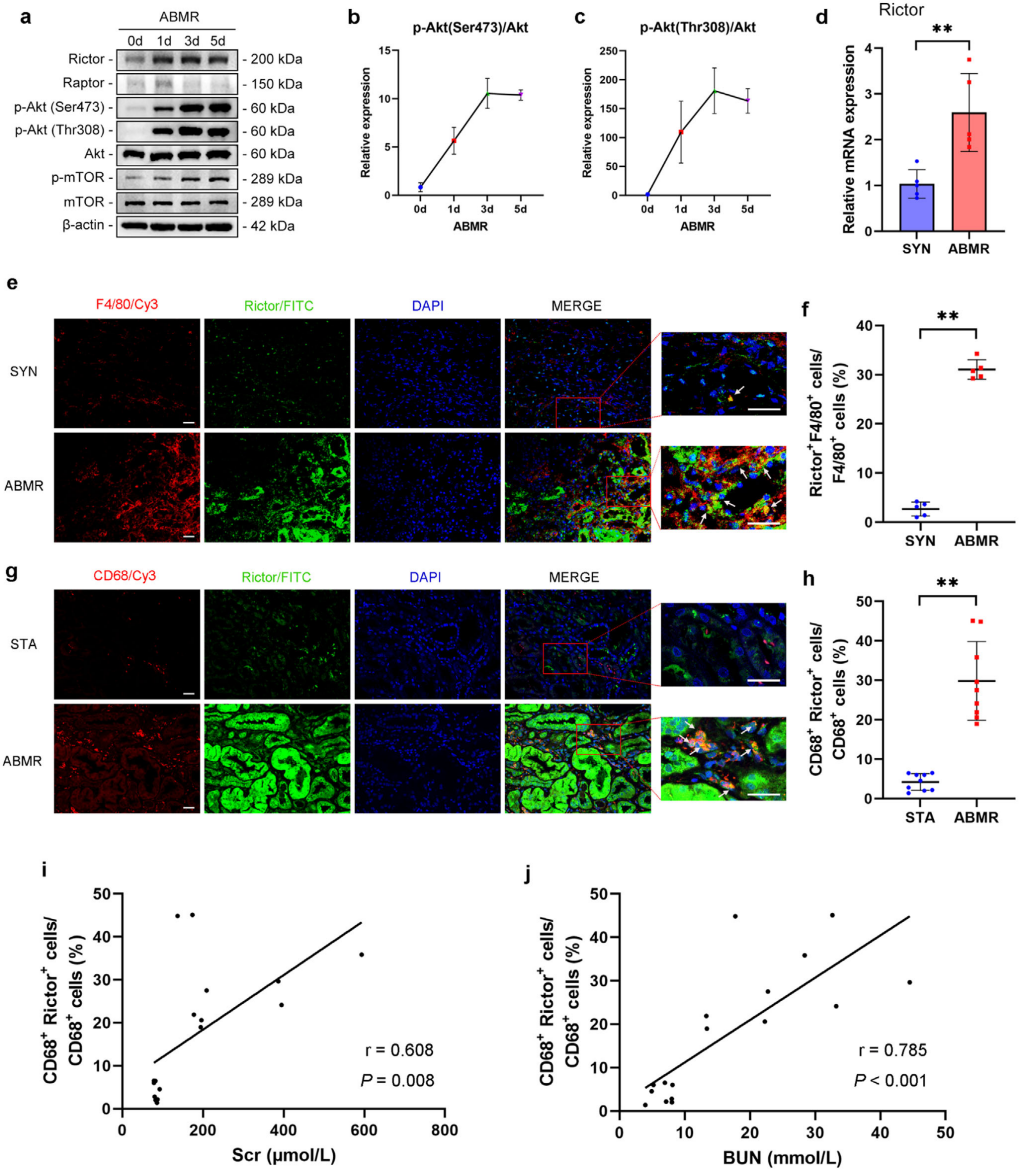
Rictor is upregulated in macrophages from mice and human kidney grafts. a–c) Western blotting assay and graphic presentation showing the induction of Rictor/mTORC2 pathway in ABMR grafts (*n* = 3). d) qRT‐PCR analysis showing the mRNA level of *Rictor* in grafts (*n* = 5). e,f) Representative IF staining and quantitative analysis for F4/80 and Rictor in grafts. White arrows indicate co‐staining‐positive cells. Original magnification × 400, Bar = 20 µm, *n* = 5. g,h) Representative IF staining and quantitative analysis for CD68 and Rictor in kidney graft biopsies from stable (STA) and ABMR patients. White arrows indicate co‐staining‐positive cells. Original magnification ×400, Bar = 20 µm, *n* = 9. i,j) The linear correlations between the expression of Rictor in macrophages and serum creatinine (Scr) and blood urea nitrogen (BUN) in STA and ABMR patients (*n* = 9). Data in (d,f,h) were analyzed using unpaired Student's *t*‐test. Data in (i,j) were analyzed using Pearson correlation coefficient. * p < 0.05; ** *p* < 0.01. Data are presented as mean ± SD.

Western blotting analysis revealed that Rictor abundance was markedly increased in ABMR grafts of mice. Following the induction of Rictor, the abundances of p‐Akt (Ser473) and p‐Akt (Thr308) increased continuously over time (**Figure** **2**a–c). The mRNA level of *Rictor* in ABMR grafts increased significantly, compared to syngeneic grafts (Figure 2d). Co‐staining of F4/80 and Rictor showed upregulation of Rictor in macrophages from ABMR grafts (Figure 2e). Among F4/80‐positive macrophages, about 29–34% of them were Rictor staining positive (Figure 2f). The sections were stained with antibodies against CD68 and Rictor (Figure 2g), and about 14–55% of CD68 positive macrophages were Rictor staining positive (Figure 2h), which is correlated with declines in graft function as analyzed by levels of serum creatinine (Scr; r = 0.608; *p* = 0.008) and blood urea nitrogen (BUN; r = 0.785; *p* < 0.001; Figure 2i,j), indicating an association of Rictor in macrophages with the severity of graft injury.

### 2.3 Rictor Deficiency in Macrophages Worsens Acute ABMR and Shortens Allografts Survival

**Figure 3:**
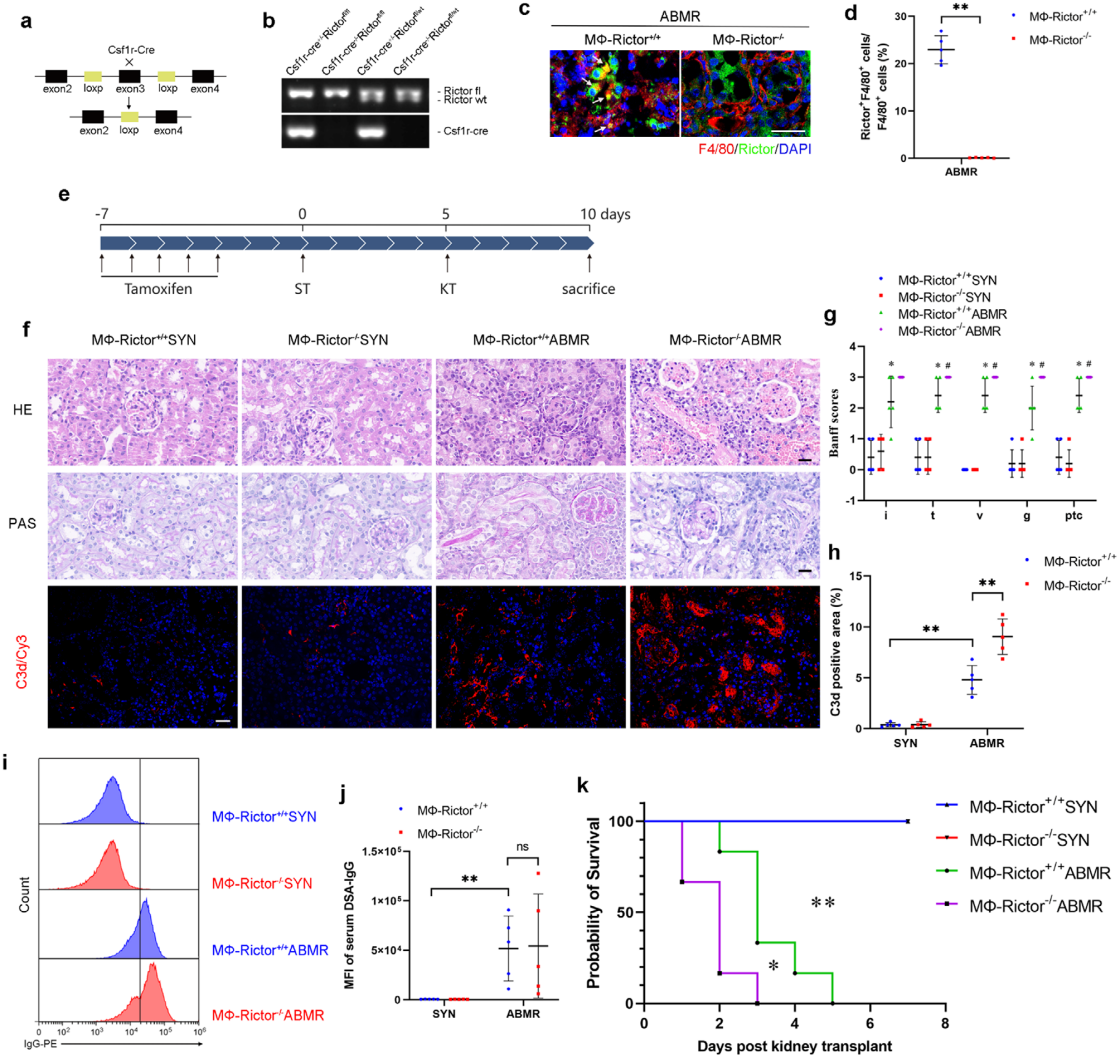
Rictor deficiency in macrophages worsens acute ABMR and shortens allografts survival. Rictor conditional knockout (MФ‐Rictor^‐/‐^) mice and their control littermates were subjected to kidney transplantation and sacrificed 5 days after operation. a) Diagram illustrates the strategy of deleting Rictor in macrophages. b) PCR analysis for genotyping mice. Lane 1: Csf1r‐Cre^+/‐^, *Rictor* ^fl/fl^; Lane 2: Csf1r‐Cre^‐/‐^, *Rictor* ^fl/fl^; Lane 3: Csf1r‐Cre^+/‐^, *Rictor* ^fl/wt^; Lane 4: Csf1r‐Cre^‐/‐^, *Rictor* ^fl/wt^. c,d) Representative IF staining and quantitative analysis for F4/80 and Rictor in ABMR allografts. White arrows indicate co‐staining‐positive cells. Original magnification ×400, Bar = 20 µm, *n* = 5. e) Strategies for tamoxifen administration and kidney transplantation in MФ‐Rictor^‐/‐^ mice and their control littermates. ST: skin transplantation; KT: kidney transplantation. f) Representative micrographs for H&E, PAS and C3d in kidney grafts. Original magnification ×400. Bar = 20 µm. g) Banff scores of interstitis, tubulitis, vasculitis, glomerulitis, peritubular capillaritis in grafts among groups as indicated. * Versus MФ‐Rictor^+/+^ SYN group; # Versus MФ‐Rictor^+/+^ ABMR group. * *p* < 0.05, *n* = 5; # *p* < 0.05, *n* = 5. h) Graphic presentation showing the C3d‐staining‐positive area in grafts among groups as indicated (*n* = 5). i,j) Flow cytometry and quantitative analysis showing the MFI of serum DSA‐IgG in the indicated groups (*n* = 5). k) Survival analysis of kidney grafts among groups as indicated, HR = 7.97, 95% CI [1.44, 43.98], *n* = 6. Data in (d,g,h,j) were analyzed using unpaired Student's *t*‐test. Data in (k) were analyzed using Gehan–Breslow–Wilcoxon test. * p < 0.05; ** *p* < 0.01. Data are presented as mean ± SD.

To explore the role of macrophage Rictor induction in kidney grafts injury, we generated a mouse model with inducible macrophage Rictor deficiency by utilizing the Cre‐LoxP system (**Figure** **3**a). Csf1r‐Cre^+/−^, *Rictor* ^fl/fl^ mice (Figure 3b, Lane 1), and Csf1r‐Cre^−/−^, *Rictor* ^fl/fl^ mice (Figure 3b, Lane 2) were subjected to kidney transplantation, and tamoxifen was injected intraperitoneally as indicated (Figure 3e). Immunofluorescence staining showed successful deletion of Rictor in macrophages from MФ‐Rictor^−/−^ mice (Figure 3c,d). Rictor deficiency in macrophages exacerbated tubular injury, thrombotic microangiopathy, glomerulitis, and peritubular capillaritis in ABMR allografts (Figure 3f,g). Notably, the absence of Rictor promoted C3d deposition in ABMR allografts (Figure 3f,h) but had no impact on serum DSA‐IgG (Figure 3i,j). Survival analysis showed that Rictor deficiency shortened ABMR allografts survival (Figure 3k). Together, these results suggest that Rictor in macrophages attenuates ABMR‐induced grafts injury and prolongs kidney allografts survival.

### 2.4 Rictor Deficiency Facilitates Macrophage M1 Polarization In Vivo and In Vitro

**Figure 4:**
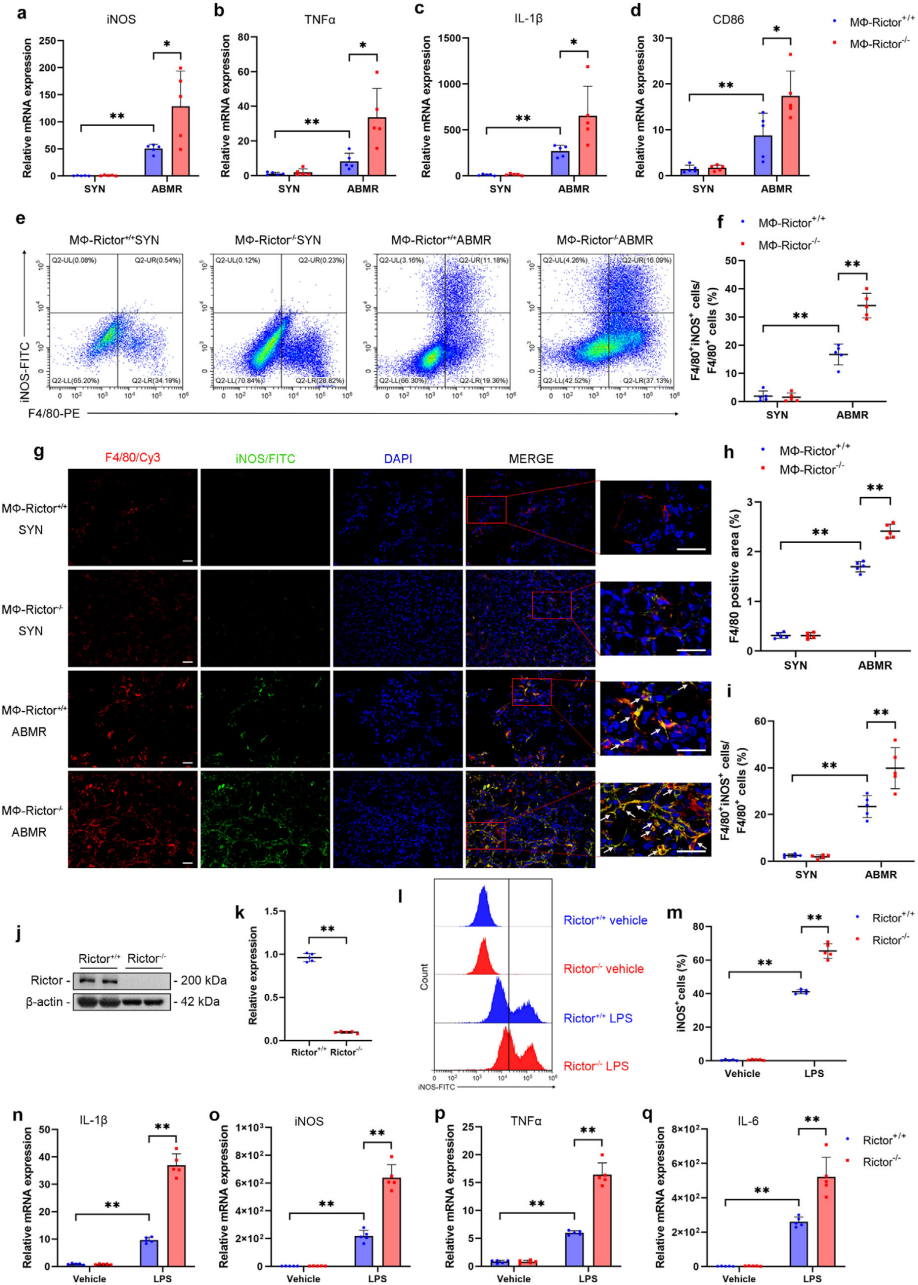
Rictor deficiency facilitates macrophage M1 polarization in vivo and in vitro. a–d) qRT‐PCR analysis showing the mRNA levels of *iNOS* (a), *TNFα* (b), *IL‐1β*(c), and *CD86* (d) in grafts among groups as indicated (*n* = 5). e,f) Flow cytometry showing quantitative analysis of the proportion of F4/80^+^ iNOS^+^ M1 macrophages in grafts among groups as indicated (*n* = 5). g–i) Representative IF staining and quantitative analysis for F4/80 and iNOS in grafts among groups as indicated. White arrows indicate co‐staining‐positive cells. Original magnification ×400, Bar = 20 µm, *n* = 5. j,k) Western blot analyses showing the deficiency of Rictor in Rictor^‐/‐^ BMDMs (*n* = 5). l,m) Flow cytometry showing the proportion of iNOS^+^ macrophages in Rictor^+/+^ and Rictor^‐/‐^BMDMs treated with or without LPS (50 ng mL^−1^) for 24 h (*n* = 5). n–q) qRT‐PCR analysis showing the mRNA levels of *IL‐1β* (n), *iNOS* (o), *TNFα* (p), and *IL‐6* (q) in BMDMs among groups as indicated (*n* = 5). Data were analyzed using unpaired Student's *t*‐test. * p < 0.05; ** *p* < 0.01. Data are presented as mean ± SD.

**Figure 5:**
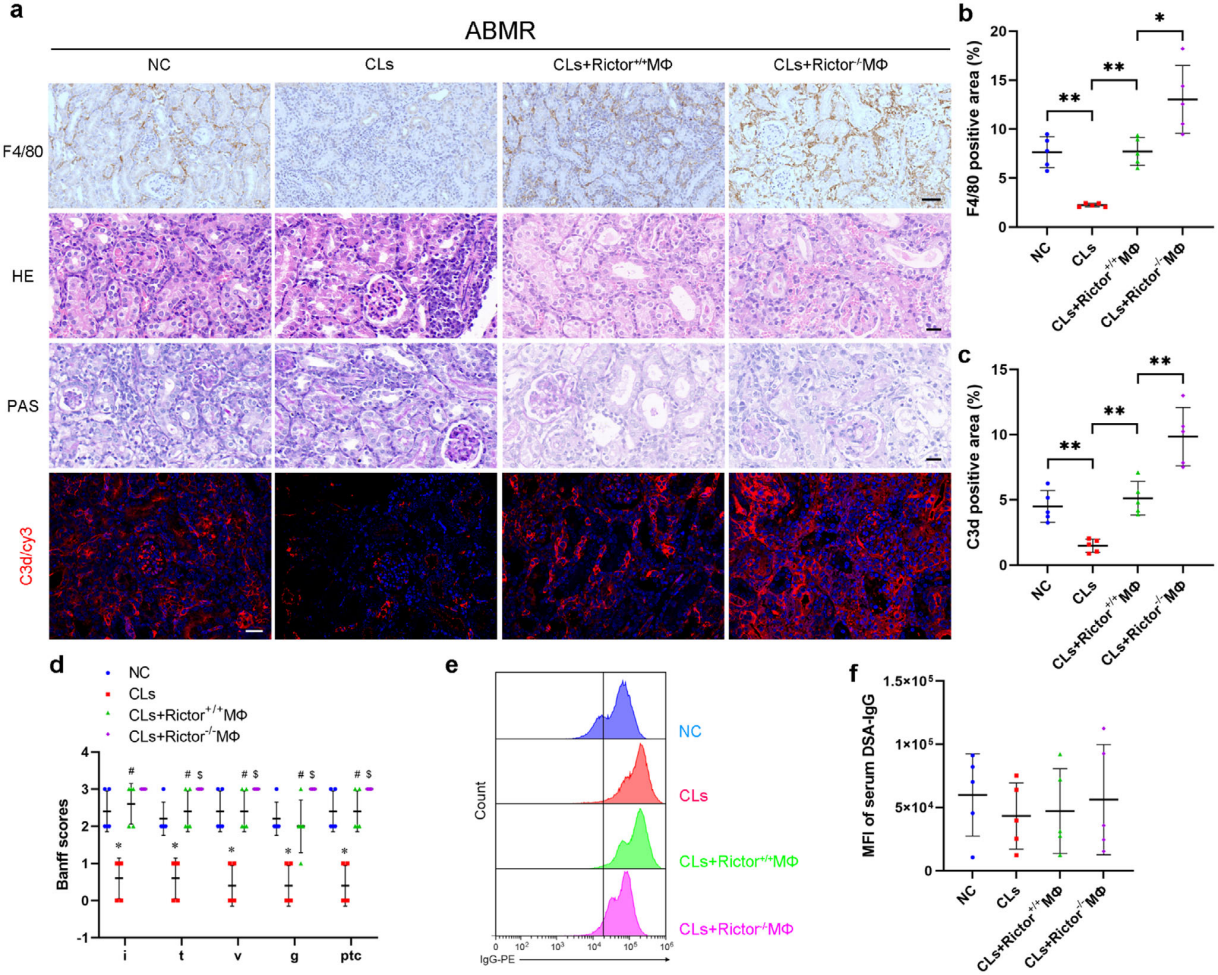
Adoptively transferred Rictor‐deficient macrophages promote acute ABMR. a) Representative immunohistochemistry for F4/80 in grafts among groups as indicated. Original magnification ×200. Bar = 50 µm. Representative micrographs for H&E, PAS, and C3d in grafts. Original magnification ×400. Bar = 20 µm. b) Quantitative analysis for F4/80 positive area in grafts (*n* = 5). c) Quantitative analysis for C3d positive area in grafts (*n* = 5). d) Banff scores of interstitis, tubulitis, vasculitis, glomerulitis, peritubular capillaritis in grafts. * Versus negative control (NC) group, *p* < 0.05, *n* = 5, # Versus Clodronate liposomes (CLs) group, *p* < 0.05, *n* = 5, $ Versus CLs+Rictor^+/+^MФ group, *p* < 0.05, *n* = 5. e,f) Flow cytometry and quantitative analysis showing the MFI of serum DSA‐IgG among groups as indicated (*n* = 5). Data were analyzed using unpaired Student's *t*‐test. * p < 0.05; ** *p* < 0.01. Data are presented as mean ± SD.

To explore the mechanisms of macrophage Rictor deficiency in exacerbating kidney grafts injury, we examined the M1‐characterized markers in kidney grafts. Compared with those in the MФ‐Rictor^+/+^ group, the mRNA levels of *iNOS*, *TNFα*, *IL‐1β* and *CD86* increased significantly in the MФ‐Rictor^‐/‐^ kidney allografts (**Figure** **4**a–d). Subsequently, flow cytometry analysis showed a higher proportion of iNOS^+^ M1 macrophages in the F4/80^+^ macrophages from the MФ‐Rictor^‐/‐^ kidney allografts, compared to the MФ‐Rictor^+/+^ kidney allografts (Figure 4e,f). Consistently, co‐staining of F4/80 and iNOS showed a deeper infiltration of F4/80^+^ macrophages and M1 phenotype (F4/80^+^ iNOS^+^) in the MФ‐Rictor^‐/‐^ kidney allografts (Figure 4g–i). To further prove the roles of Rictor in macrophage activation, we cultured bone‐marrow‐derived macrophages (BMDMs) and validated the ablation of Rictor (Figure 4j,k). Compared to BMDMs derived from MФ‐Rictor^+/+^ mice, those from MФ‐Rictor^‐/‐^ mice showed a higher content of LPS‐induced iNOS^+^ M1 macrophages (Figure 4l,m). Additionally, Rictor deficiency boosted the mRNA levels of LPS‐induced *iNOS*, *TNFα*, *IL‐1β*, *IL‐6 in vitro* (**Figure** **5**n–q). Taken together, these observations support an indispensable role for Rictor in macrophage M1 polarization.

### 2.5 Adoptively Transferred Rictor‐Deficient Macrophages Promote Acute ABMR

To elucidate the potential mechanism of macrophage Rictor in ABMR, we depleted macrophages using Clodronate liposomes (CLs). Treatment with CLs significantly reduced F4/80^+^ macrophage infiltration, C3d deposition, and histological injury in ABMR allografts (Figure 5a–d). Adoptive transfer of Rictor^+/+^ macrophages into the mice significantly rescued the effects of CLs on ABMR, but transfer of Rictor^‐/‐^ macrophages led to worse histological injury and elevated macrophages infiltration and C3d deposition (Figure 5a–d). However, both transfers had no influence on the level of serum DSA‐IgG (Figure 5e,f). Together, these results support a role of macrophages, specifically Rictor‐expressing macrophages, in acute ABMR.

### 2.6 Rictor Suppresses Macrophage M1 Polarization by Inhibiting NLRP3 Inflammasome Activation

**Figure 6:**
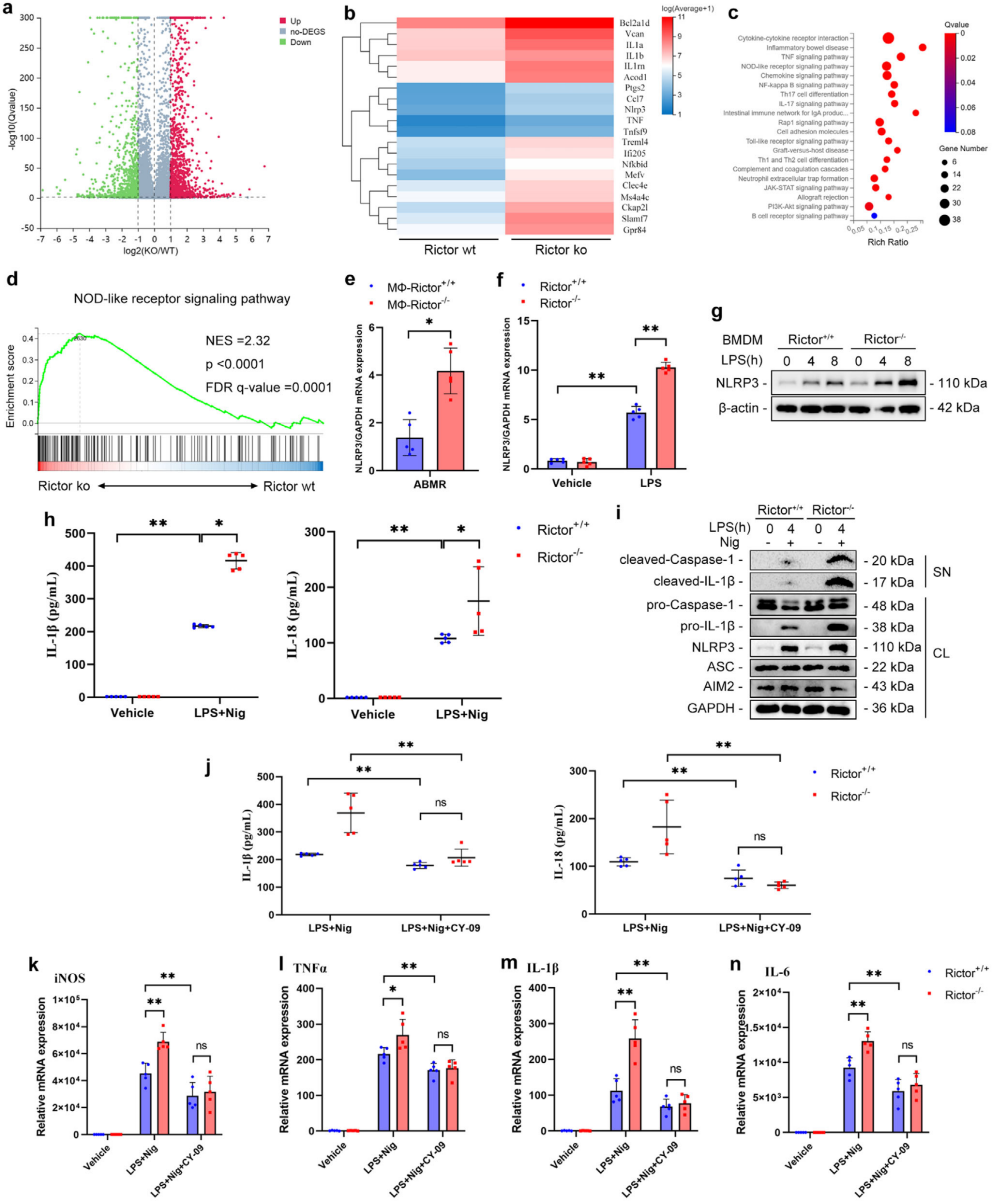
Rictor suppresses macrophage M1 polarization by inhibiting NLRP3 inflammasome activation. a) The volcano plot showing differential expression genes (DEGs) between macrophages in MФ‐Rictor^+/+^ and MФ‐Rictor^‐/‐^ ABMR grafts (*n* = 3). b) Expression heatmap showing top 20 DEGs relative to macrophage polarization (*n* = 3). c) KEGG analysis showing top 20 pathways between macrophages in MФ‐Rictor^+/+^ and MФ‐Rictor^‐/‐^ ABMR grafts (*n* = 3). d) Gene Set Enrichment Analysis (GSEA) of the NOD‐like receptor signaling pathway gene set between macrophages in MФ‐Rictor^+/+^ and MФ‐Rictor^‐/‐^ ABMR grafts (*n* = 3). e) qRT‐PCR analysis showing the mRNA level of *NLRP3* in macrophages of ABMR grafts (*n* = 3). f) qRT‐PCR analysis showing the mRNA level of *NLRP3* in WT or Rictor^‐/‐^ BMDMs after priming with LPS (200 ng mL^−1^) for 4 h (*n* = 5). g) Western blotting analysis of NLRP3 in WT or Rictor^‐/‐^ BMDMs after priming with LPS (200 ng mL^−1^) for indicated time. h) Enzyme‐linked immunosorbent assay (ELISA) analysis of IL‐1β and IL‐18 in the supernatant of WT or Rictor^‐/‐^ BMDMs following priming with LPS (200 ng mL^−1^) for 4 h and subsequent stimulation with nigericin (50 µm) for 30 min (*n* = 5). i) Western blotting analysis of the indicated proteins in LPS‐primed BMDMs treated with nigericin (50 µm). j) ELISA analysis of IL‐1β and IL‐18 in the supernatant of LPS (200 ng mL^−1^)‐primed BMDMs treated with CY‐09 (5 µm) and subsequently stimulated with nigericin (50 µm) (*n* = 5). k–n) qRT‐PCR analysis showing the mRNA levels of *iNOS* (k), *TNFα* (l), *IL‐1β* (m), and *IL‐6* (n) in LPS (200 ng mL^−1^)‐primed BMDMs from WT or Rictor^‐/‐^ mice treated with CY‐09 (5 µm) and subsequent stimulated with nigericin (50 µm) among groups as indicated (*n* = 5). Data for bar charts were analyzed using unpaired Student's *t*‐test. * p < 0.05; ** *p* < 0.01. Data are presented as mean ± SD.

To explore the mechanisms of macrophage Rictor in regulating macrophage M1 polarization, CD115^+^ monocytes/macrophages were sorted from ABMR grafts by microbeads and subjected to RNA sequencing. As illustrated in the volcano plot, 1159 genes were markedly upregulated, and 850 genes were downregulated in MФ‐Rictor^‐/‐^ mice versus MФ‐Rictor^+/+^ mice (**Figure** **6**a). The hierarchical clustering heatmap displayed the top 20 differentially expressed genes (DEGs) relative to macrophage polarization (Figure 6b). The KEGG analysis showed that multiple inflammatory‐related signaling pathways were significantly upregulated in the MФ‐Rictor^‐/‐^ kidney allografts (Figure 6c). Gene Set Enrichment Analysis (GSEA) revealed that Rictor expression in macrophages of ABMR grafts was negatively correlated with the activity of the NOD‐like receptor signaling pathway (Figure 6d). The mRNA level of *NLRP3* in the CD115^+^ monocytes/macrophages sorted from ABMR grafts was validated (Figure 6e). Consistently, the mRNA and protein levels of NLRP3 increased markedly by Rictor deficiency in the BMDMs treated with LPS (Figure 6f,g). Additionally, the concentrations of IL‐1β and IL‐18 in the supernatant increased significantly in Rictor‐deficient BMDMs treated with LPS and nigericin. Nig‐stimulated IL‐1β secretion and caspase‐1 cleavage rose markedly in LPS‐primed Rictor‐deficient BMDMs (Figure 6h,i). Rictor deficiency had no effect on the expression of AIM2, another pattern recognition receptor, which could form inflammasomes (Figure 6i). Moreover, CY‐09, the inhibitor of NLRP3 activation by bounding to the adenosine triphosphate binding site of the NLRP3 NACHT domain,^[^ ^20^ ^]^ markedly inhibited the production of IL‐1β and IL‐18 induced by Rictor deficiency (Figure 6j). Notably, Rictor‐mediated expression of M1‐characterized markers, including *iNOS*, *TNFα*, *IL‐1β*, and *IL‐6*, was suppressed by CY‐09 (Figure 6k–n). In summary, these results indicate that Rictor suppresses macrophage M1 polarization by inhibiting NLRP3 inflammasome activation.

### 2.7 Rictor Inhibits NLRP3 Transcription Through Increasing the K48‐Linked Ubiquitination of p65 by E3 Ubiquitin Ligase SOCS1

**Figure 7:**
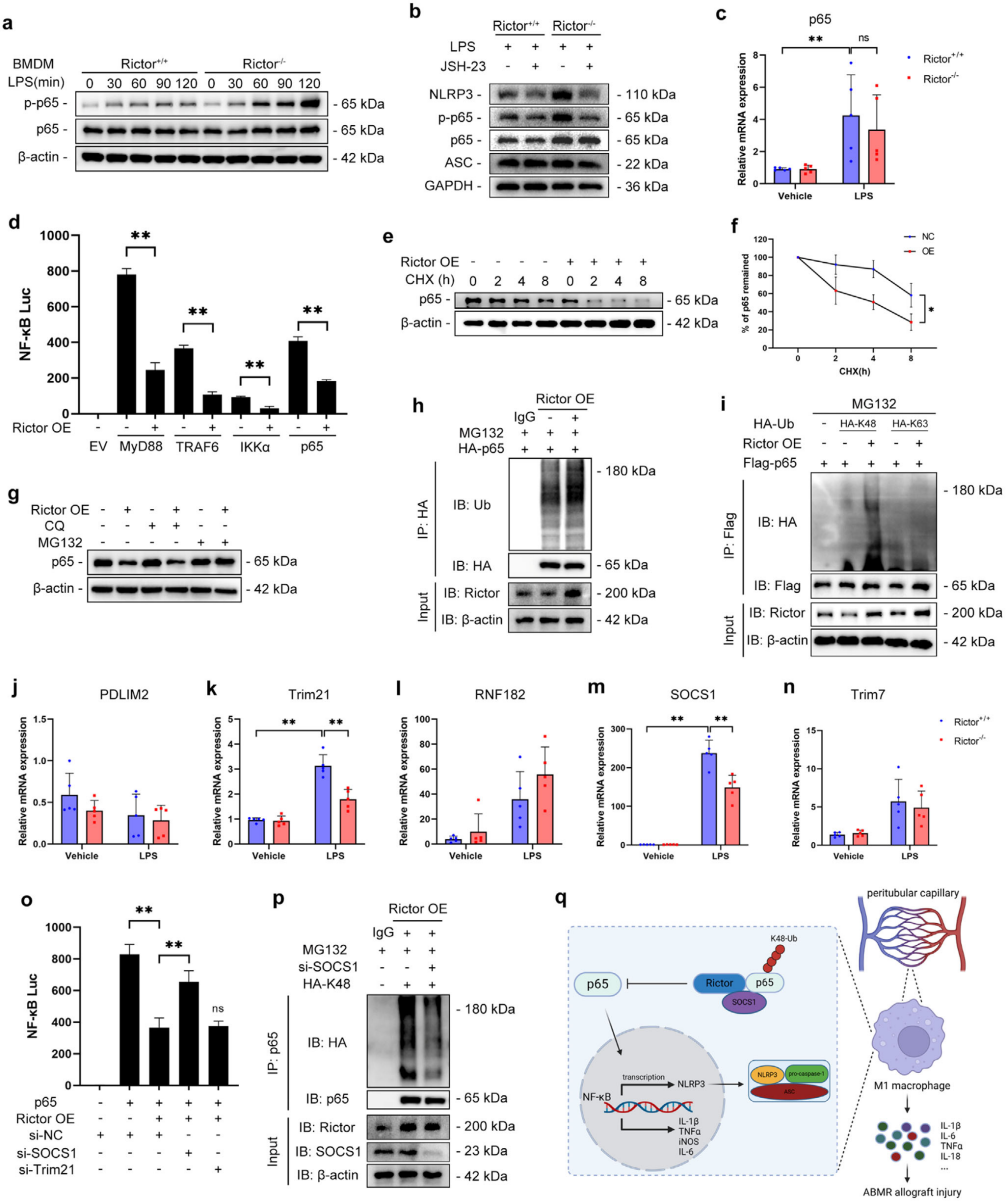
Rictor inhibited NLRP3 transcription through increasing the K48‐linked ubiquitination of p65 by E3 ubiquitin ligase SOCS1. a) Western blotting analysis of total and phosphorylated p65 in WT or Rictor^‐/‐^BMDMs treated with LPS (200 ng mL^−1^) for indicated time. b) Western blotting analysis of indicated proteins in WT or Rictor^‐/‐^BMDMs priming with LPS (200 ng mL^−1^) and JSH‐23 (20 µm) for 4 h. c) qRT‐PCR analysis showing the mRNA level of *p65* in WT or Rictor^‐/‐^ BMDMs treated with LPS (200 ng mL^−1^) for 4 h (*n* = 5). d) Luciferase activity in negative control (NC) or Rictor overexpression (OE) HEK293T cells transfected with plasmids encoding an NF‐κB luciferase reporter and TK‐Renilla reporter, together with indicated plasmids, was measured at 24 h after transfection and normalized to the Renilla luciferase activity (*n* = 3). e) Western blotting analysis of p65 in NC and Rictor OE HEK293T cells were treated with CHX (200 µg mL^−1^) for indicated time. f) Decay curve of p65 normalized to β‐actin and to 0 h at indicated time points from CHX‐chase assay (*n* = 3). g) Western blotting analysis of p65 in NC or Rictor OE HEK293T cells transfected with Flag‐p65 and treated with MG132 (10 µm) for 6 h or CQ (10 µm) for 24 h. h) Co‐immunoprecipitation (IP) assay for the ubiquitination of p65 in NC or Rictor OE HEK293T cells transfected with HA‐p65 and treated by MG132. i) Co‐IP assay for the ubiquitination of p65 in NC or Rictor OE HEK293T cells transfected with the indicated plasmids and treated by MG132. j–n) qRT‐PCR analysis showing the mRNA levels of *PDLIM2* (j), *Trim21* (k), *RNF182* (l), *SOCS1* (m) and *Trim7* (n) in WT or Rictor^‐/‐^ BMDMs treated with LPS (200 ng mL^−1^) for 4 h (*n* = 5). o) HEK293T cells transfected with plasmids encoding an NF‐κB luciferase reporter and TK‐Renilla reporter, together with Flag‐p65 and indicated siRNAs, were measured at 24 h after transfection and normalized to the Renilla luciferase activity (*n* = 3). p) Co‐IP assay for the ubiquitination of p65 in NC or Rictor OE HEK293T cells transfected with the HA‐K48 and si‐SOCS1 and treated by MG132. q) A schematic model for this study. Data were analyzed using unpaired Student's *t*‐test. * *p* < 0.05; ** *p* < 0.01. Data are presented as mean ± SD.

Considering that Rictor deficiency promoted the mRNA levels of LPS‐induced *iNOS*, *TNFα*, *IL‐1β*, *IL‐6*, and *NLRP3*, Rictor may regulate the priming process of NLRP3 inflammasome activation. The transcription factor NF‐κB is critical for NLRP3 expression. To elucidate the mechanism enhancing NF‐κB activity in Rictor‐deficient BMDMs, we examined total protein and phosphorylation levels of core kinases involved in canonical NF‐κB signaling. We found that compared to WT cells, p‐p65 were enhanced in Rictor‐deficient BMDMs. However, the total protein level of p65 increased in response to Rictor deficiency (**Figure** **7**a,b). JSH‐23, an inhibitor of NF‐κB, suppressed the expression of NLRP3, suggesting that p65 is required for the transcription of NLRP3 (Figure 7b). However, *p65* mRNA level did not change significantly in Rictor‐deficient BMDMs (Figure 7c). Subsequently, NF‐κB luciferase activity was markedly activated by overexpression of MyD88, TRAF6, IKKα, and p65, but all their activities were inhibited when Rictor was overexpressed, suggesting that Rictor may block NF‐κB activation at the downstream signaling level of p65 (Figure 7d). Furthermore, CHX‐chase assay showed that the degradation rate of p65 increased in Rictor‐OE cells, compared to WT cells (Figure 7e,f). To investigate whether Rictor degrades p65 through an autolysosome or proteasome pathway, HEK293T cells were transfected with p65 and treated with different pharmacological inhibitors. The degradation of p65 induced by Rictor was blocked by proteasome inhibitor MG132, but not by autophagy inhibitor chloroquine (CQ) (Figure 7g). Co‐IP assay showed that Rictor overexpression led to an increase of p65 ubiquitination (Figure 7h). To ascertain the ubiquitination modification of p65 affected by Rictor, we transfected either K48‐linked or K63‐linked ubiquitination plasmids into HEK293T cells. The results indicated that Rictor overexpression increased the K48‐linked ubiquitination of p65, leaving K63‐linked ubiquitination unaffected (Figure 7i). To investigate the specific E3 ubiquitin ligase regulating Rictor‐medicated degradation of p65, we used the UbiBrowser platform (http://ubibrowser.ncpsb.org) to predict the specific E3 ubiquitin ligases interacting with p65. *Trim21* and *SOCS1* were downregulated by Rictor deficiency (Figure 7j–n). Luciferase assay in Rictor‐OE HEK293T cells transfected with scramble, Trim21 or SOCS1 siRNA suggested that Rictor‐mediated inhibition of NF‐κB activation was blocked by knocking down SOCS1, but not Trim21 (Figure 7o). Notably, SOCS1 knockdown alleviated the K48‐linked ubiquitination degradation of p65 in the presence of Rictor (Figure 7p). The lysine residues K28 and K195 were predicted as potential ubiquitination sites by GPS‐Uber (https://gpsuber.biocuckoo.cn/). The two p65 mutant plasmids with individual lysine residues substituted by arginine. The following co‐IP assay demonstrated that the K195R mutant showed reduced ubiquitination, suggesting that the lysine residue K195 is crucial for the p65 ubiquitination mediated by Rictor (Figure S2a, Supporting Information).To clarify the binding site between p65 and SOCS1, full‐length (HA‐FL) and truncated plasmids [HA‐SOCS1‐N (amino acids 1–78, T1), HA‐SOCS1‐SH2 (amino acids 79–174, T2), HA‐SOCS1‐C (amino acids 175–211, T3)] based on the structural domain sequences of SOCS1 were constructed (Figure S2b, Supporting Information). The co‐IP assay illustrated p65 selectively binds to the SH2‐domain of SOCS1 (Figure S2c, Supporting Information). SOCS1 overexpression prevented Rictor‐deficient macrophages from LPS‐induced elevated polarization markers of M1 macrophages (Figure S2d, Supporting Information). Taken together, these results suggest that Rictor inhibits NLRP3 transcription through increasing the K48‐linked ubiquitination of p65 by E3 ubiquitin ligase SOCS1.

## 3 Discussion

Here we report that Rictor deficiency in macrophages promotes M1 polarization, thereby exacerbating ABMR injury and shortening the survival of kidney grafts. At the molecular level, the ablation of Rictor inhibits the expression of E3 ubiquitin ligase SOCS1, decreased the ubiquitination and degradation of p65, and then upregulates the activity of NF‐κB and the expression of NLRP3, thus contributing to the overproduction‐of‐inflammatory cytokines, such as IL‐1β and TNFα. Our study is the first to reveal the unique role and mechanism of macrophage Rictor in ABMR.

Macrophages play a critical role in the acute rejection following kidney transplantation.^[^ ^21^ ^]^ They can serve as either immune cells integral to innate immunity, or antigen‐presenting cells to induce adaptive immune responses.^[^ ^22^ ^]^ A significant heterogeneity shows up among macrophage phenotypes in kidney transplant rejection. Under different immune microenvironments and stimuli, macrophages can polarize into M1 macrophages, which primarily promote inflammatory responses, or M2 macrophages, which are involved in cell proliferation and tissue repair.^[^ ^12^, ^23^, ^24^ ^]^ Through secreting various cytokines, macrophages are essential in maintaining immune homeostasis. Previous studies have shown that macrophage infiltration in glomerular capillaries during acute and chronic rejection is associated with ABMR and predicts subsequent transplant failure.^[^ ^10^ ^]^ Furthermore, early macrophage infiltration in renal biopsies of allografts correlates with subsequent interstitial fibrosis.^[^ ^9^ ^]^ Our work is the first to demonstrate that Rictor/mTORC2 modulates macrophage polarization by regulating p65‐NLRP3 axis in the immune microenvironment of ABMR allografts. However, the macrophage biology in human biopsy samples surely differs from that in murine models at various levels. Thus, the findings from model mice cannot yet be directly extrapolated to humans, and should be further validated before clinical translation. Existing therapeutic strategies, such as complement inhibitors and B‐cell depletion, focus on B‐cell activation and antibody production, showing limited outcome. Our study confirmed that the specific regulation of macrophages is independent of antibody generation, providing new therapeutic strategies against ABMR.

mTOR1 inhibitors have been introduced into immunosuppressive therapy for kidney transplantation, but their application remains limited.^[^ ^25^ ^]^ The TRANSFORM study indicated that an immunosuppressive regimen comprising everolimus, low‐dose tacrolimus, and prednisone is not inferior to the classic tacrolimus‐mycophenolate mofetil‐prednisone regimen.^[^ ^26^ ^]^ However, the debate is still ongoing regarding the role of mTOR1 inhibitors in kidney transplantation. Our study found that Rictor and its downstream kinases, rather than the Raptor/mTORC1 signaling pathway, were significantly activated in ABMR. In response to TLR stimulation, the mTORC2 signaling pathway is activated by upstream PI3K through directly phosphorylating Akt at Ser473 and Thr308,^[^ ^15^ ^]^ which is consistent with what we observed. It has been reported that Akt1‐deficient macrophages exhibited increased cytokine production, and Akt1‐deficient mice showed impaired tolerance to LPS stimulation,^[^ ^27^ ^]^ partially explaining the effects of Rictor loss on macrophage M1 polarization. Nevertheless, how to enable Rictor‐specific modulation, meanwhile avoiding off‐target effects, remains a thorny question. The agonists targeting Rictor have not been developed yet. No significant difference in weight and survival rate was found in macrophage Rictor knockout mice. While our data are promising, its efficacy and safety remain unproven in humans. In biopsy samples from ABMR patients, Rictor was significantly upregulated in macrophages, which is correlated with the severity of kidney injury. Therefore, the Rictor/mTORC2 signaling pathway may be targeted to design new anti‐ABMR therapies.

NOD‐like receptors serve as intracellular innate immune receptors that coordinate inflammatory responses through inflammasome assembly.^[^ ^28^ ^]^ Our study revealed a significant correlation between Rictor deficiency and the activation of the NOD‐like receptors signaling pathway in ABMR transplanted kidneys, with a marked increase in NLRP3 transcription levels, suggesting that Rictor‐mediated macrophage M1 polarization and kidney injury may be associated with NLRP3 inflammasome activation. By employing CY‐09 to inhibit NLRP3 inflammasome activation,^[^ ^20^ ^]^ we found that CY‐09 significantly suppressed Rictor‐mediated macrophage M1 polarization and inflammatory cytokine secretion. Consistently, microbial metabolite TMAO was found to activate the NLRP3 inflammasome, the deletion of which in BMDMs impeded TMAO‐induced M1 polarization.^[^ ^29^ ^]^ As one of the pattern recognition receptors in the innate immune system, NLRP3 is widely regulated by post‐translational modifications (PTMs).^[^ ^30^ ^]^ Among them, the ubiquitination of NLRP3 is crucial for the assembly of inflammasome complexes.^[^ ^31^ ^]^ Multiple E3 ubiquitin ligases promote NLRP3 ubiquitination and protein degradation, thereby limiting NLRP3 inflammasome activity. However, our study found that the absence of Rictor significantly upregulated the transcriptional and protein levels during NLRP3 inflammasome activation, suggesting that Rictor is not involved in PTM regulation of NLRP3. Furthermore, NF‐κB is critical for the transcription of NLRP3 and inflammatory factors.^[^ ^32^, ^33^ ^]^ Therefore, we investigated the changes in the upstream NF‐κB signaling pathway of NLRP3, and found that consistent with transcriptomic sequencing results, the NF‐κB signaling pathway was significantly activated, while the transcriptional level of its key subunit p65 remained unaffected, suggesting that p65 might be regulated by PTMs. In fact, we verified that Rictor promoted the degradation of p65 through the proteasome pathway, rather than the autophagy pathway. SOCS1, crucial among the SOCS family, inhibits inflammation by promoting JAK ubiquitination and suppressing STAT phosphorylation.^[^ ^34^, ^35^ ^]^ Our study found that knocking down SOCS1 negates the Rictor‐mediated inhibition of NF‐κB activation and p65 degradation.

In conclusion, Rictor modulates macrophage M1 polarization through the p65‐NLRP3 axis, thereby alleviating ABMR‐induced kidney injury and prolonging the survival of grafts. These results provide potential strategies for the prevention and treatment of ABMR following kidney transplantation.

## 4 Experimental Section

### Human Subjects

This study was approved by the Ethics Committee of the Second Affiliated Hospital of Nanjing Medical University (approval number: 2022‐KY‐116‐01). The sample of kidney graft biopsies from stable and ABMR patients were obtained from the Second Affiliated Hospital of Nanjing Medical University. ABMR was diagnosed according to Banff 2019.^[^ ^4^ ^]^ The informed consent was obtained from all human subjects. The techniques used in this study adhered to the Helsinki and Istanbul Declarations’ Ethical standards.

### Mice

Male C57BL/6 (H‐2^b^) and BALB/c (H‐2^d^) mice, weighing 20–25 g, were obtained from Nanjing Medical University. All mice were housed in groups of five per cage in a room with a 12‐h light/dark cycle, maintained at a temperature of 20–26 °C with a relative humidity of 40–60%. All the experimental procedures were approved by the Institutional Animal Care and Use Committee at Nanjing Medical University (IACUC‐2109025).

Csf1r‐Cre transgenic mice and Rictor floxed mice on C57BL/6J background were kindly provided by Professor Chunsun Dai from the Center for Kidney Disease of the Second Affiliated Hospital of Nanjing Medical University.^[^ ^17^ ^]^ By mating Rictor floxed mice with Csf1r‐Cre transgenic mice, mice with genotyping Csf1r‐Cre^+/−^, Rictor^fl/fl^, and the same gender littermates with genotyping Csf1r‐Cre^−/−^, Rictor^fl/fl^ were generated and then randomly divided into different groups. Male mice at 8 weeks of age were intraperitoneally injected with tamoxifen (#T5648, Sigma–Aldrich) at 75 mg kg^−1^ for 5 consecutive days to induce *Rictor* gene ablation.

At day 7 after the last administration, full‐thickness skin grafts (1.0 cm × 1.0 cm) from the backs of BALB/c donor mice were transplanted onto the backs of C57BL/6 recipient mice. At day 5 after skin transplant, kidney transplantation was carried out on C57BL/6 mice, who received a kidney from a BALB/c donor as an allograft or from a C57BL/6 donor as a syngeneic (SYN) graft. Ectopic kidney transplantation was performed as described previously.^[^ ^36^ ^]^ In brief, the left donor kidney was transplanted into the abdominal cavity of the recipient, with both of its native kidneys retained; then the artery and vein of the donor kidney were anastomosed to the recipient's abdominal aorta and vena cava using 10‐0 sutures. The donor ureter was embedded into the recipient's bladder. The technical success rate of the transplantation procedure was 90%. Efforts were made to minimize the sufferings of mice during experiments.

### Histological, Immunohistochemistry, and Immunofluorescence Staining

The kidney grafts were obtained and processed with 4% paraformaldehyde fixation and paraffin embedment. 5 µm thickness sections were stained with hematoxylin and eosin (H&E) and periodic acid Schiff (PAS), according to the standard steps. CD68 (#MA5‐12407, Invitrogen), F4/80 (#14‐4801, eBioscience), iNOS (#ab49999, Abcam), Rictor (#A300‐459A, Bethy lab) expression levels in the kidney grafts were assessed using immunohistochemistry and immunofluorescence staining.

### Flow Cytometry

The harvested kidney grafts were ground and digested in DMEM containing 1 mg mL^−1^ collagenase I (#C0130, Sigma–Aldrich) and 0.1 mg mL^−1^ DNase I (#10104159001, Roche) and then filtered through a 40‐µm mesh to get single‐cell suspension. The level of circulating anti‐donor DSAs of IgG in the recipient serum was assessed by flow cytometry. The following fluorochrome‐conjugated antibodies were used for cell staining: PE‐conjugated IgG (#406607, Biolegend), PE‐conjugated F4/80 (#14‐4801‐82, eBioscience), FITC‐conjugated iNOS (#53‐5920‐82, eBioscience), APC‐conjugated CD206 (#17‐2061‐8, eBioscience). Single‐cell suspensions were analyzed by a CytoFLEX flow cytometer (Beckman Coulter), and the obtained data were subjected to analyses using CytExpert software.

### Cell Culture and Treatment

Bone‐marrow‐derived macrophages (BMDMs) were isolated as previously described.^[^ ^19^ ^]^ Briefly, bone marrow suspension was obtained by flushing the bone marrow cavity of the femur and tibia. Monocytes were isolated from the bone marrow suspension using Lymphoprep (#MLSM1092, MULTISCIENCES Biotech). BMDMs were cultured in Dulbecco's modified Eagle's medium (DMEM) containing 10% (v/v) FBS (#04‐001‐1ACS, BIOIND), 1% (v/v) antibiotics (#15070063, Gibco) and 30 ng mL^−1^ mouse M‐CSF (#CB34; Novoprotein Scientific Inc). The culture medium was refreshed every 3 days. For Rictor overexpression, BMDMs were transfected with either empty vector lentivirus or Flag‐Rictor lentivirus (Genechem) following the manufacturer's instructions.

### Western Blotting and Coimmunoprecipitation (Co‐IP)

Total protein was extracted by RIPA lysis buffer (Beyotime, Shanghai) and the concentrations were quantified with BCA assay. For Western blotting assays, proteins were separated by SDS‐polyacrylamide gel electrophoresis and then transferred onto polyvinylidene‐fluoride membranes. The incubation was performed at 4 °C overnight with the primary antibodies against Rictor (1:1000, #2114, CST), Raptor (1:1000, #2280, CST), Akt (1:2000, #10176‐2‐AP, Proteintech), p‐Akt(Ser473) (1:1000, #66444‐1‐Ig, Proteintech), pAkt(Thr308) (1:1000, #9275, CST), mTOR (1:1000, #2983, CST), p‐mTOR (1:1000, #5536, CST), NLRP3 (1:1000, #15101, CST), IL‐1β (1:1000, #31202, CST), Caspase‐1 (1:1000, #24232, CST), cleaved‐IL‐1β (1:1000, #63124, CST), cleaved‐Caspase‐1 (1:1000, #AG‐20B‐0042, AdigoGen), ASC (1:1000, #67824, CST), AIM2 (1:1000, #63660, CST), SOCS1 (1:1000, #55313, CST), p65 (1:1000, #8242, CST), p‐p65 (1:1000, #TA325803, Origene), ubiquitin (#sc‐8017, Santa Cruz), Flag (#14793, CST), HA (#51064‐2‐AP, Proteintech), Myc (#sc‐40, Santa Cruz), GAPDH (1:10000, #60004‐1‐Ig, Proteintech) and β‐actin (1:10000, #66009‐1‐Ig, Proteintech). Following another incubation with horseradish peroxidase‐conjugated secondary antibodies, the signal was detected with an imaging system.

Co‐IP assays were performed using an IP kit (#abs955, Absin) following the supplier's instructions. Protein samples were incubated with the primary antibodies at 4 °C overnight and then mixed with Protein A/G magnetic beads at room temperature for 30 min. After magnetic separation, the protein‐beads complexes were washed with lysis buffer three times, then boiled in 1X SDS loading buffer and finally subjected to Western blotting analysis.

### Quantitative Real‐Time Polymerase Chain Reaction (qRT‐PCR)

Total RNA was extracted using the RNA Isolation Kit (#RC112‐01, Vazyme), quantified, and reverse‐transcribed using HiScript III All‐in‐one RT SuperMix Perfect for qPCR kit (#R333‐01, Vazyme). Gene expression was determined by qRT‐PCR (#Q341‐AA, Vazyme). The expression level of each mRNA was normalized to GAPDH mRNA level. Primer sequences are listed in Table S1 (Supporting Information).

### Depletion and Adoptive Transfer of Macrophages

Clodronate liposomes (#40337ES08, Yeasen) and control liposomes (#40338ES05, Yeasen) were intraperitoneally injected into C57BL/6 mice to delete macrophages. At day 2 after injection, ABMR was induced as described previously, and BMDMs (5 × 10^6^ cells per mouse) from MФ‐Rictor^‐/‐^ mice and MФ‐Rictor^+/+^ mice were adoptively transferred into the mouse immediately after reperfusion. The efficiency of macrophage depletion was confirmed by immunohistochemistry.

### CD115^+^ Monocyte/Macrophage Enrichment

The harvested kidney grafts were ground and digested in DMEM containing 1 mg mL^−1^ collagenase I and 0.1 mg mL^−1^ DNase, and then the mixture was filtered through 40‐µm mesh to get single‐cell suspension. CD115^+^ monocyte/macrophage were enriched from the single‐cell suspension with PE‐conjugated CD115 antibody (#135505, Biolegend), PE Microbeads (#17666, Stemcell), and DYNAL bead separations (Invitrogen) according to the manufacturer's instruction. RNA‐Seq libraries were prepared following the manufacturer's instructions. The significant levels were corrected by Q value with a threshold (Q value ≤ 0.05) by Benjamini, Hochberg. Differential expression analysis was performed using the DESeq2 (v1.34.0) with Q value ≤ 0.05 and fold change ≥ 1. Heatmap clustering, Kyoto Encyclopedia of Genes and Genomes (KEGG) analysis and Gene Set Enrichment Analysis (GSEA)were performed by Dr. Tom 2.0 (BGI, Shenzhen, China). The data are openly available in Genome Sequence Archive (GSA) (https://ngdc.cncb.ac.cn/), reference number CRA021234.

### Luciferase and Reporter Assays

HEK293T cells were transfected with pRL‐TK‐Renilla‐luciferase plasmid and plasmids encoding the NF‐κB luciferase reporter, together with indicated plasmids. Luciferase activity was analyzed by Dual‐Luciferase Reporter Assay Kit (#DL101‐01, Vazyme). Data were normalized for transfection efficiency by Renilla luciferase activity.

### Enzyme‐Linked Immunosorbent Assay (ELISA)

IL‐1β (#ml301814, mlbio) and IL‐18 (#ml002294, mlbio) in cell supernatants were measured following the manufacturer's instructions. Absorbance was detected at 450 nm by the Rayto RT‐6100.

### RNA Interference Assay and Plasmids

HEK293T cells were transfected with siRNA targeting SOCS1 and Trim21 using Rfect reagent. The siRNA targeting sequences were GCACCUUCCUGGUGCGCGATT (SOCS1), GCAGGAGUUGGCUGAGAAGTT (Trim21), and UUCUCCGAACGUGUCACGUTT (control).

The Myc‐Ub (P25115), HA‐Ub‐K48 (P31802), HA‐Ub‐K63 (P31800), Flag‐p65 (P45239), Flag‐MyD88 (P52168), Flag‐TRAF6 (P52168), Flag‐IKKα (P54856) and Flag‐SOCS1 (P71548) plasmids were obtained from MIAOLING BIOLOGY. HA‐Ub‐WT plasmid was sourced from Addgene. The HA‐SOCS1‐FL, HA‐SOCS1‐ N (amino acids 1–78, T1), HA‐SOCS1‐SH2 (amino acids 79–174, T2), HA‐SOCS1‐C (amino acids 175–211, T3) and site‐directed mutations of p65 (K28R, K195R) were generated by Jiangsu KeyGEN BioTECH. All the constructed plasmids were confirmed by DNA sequencing. The transfection of plasmids was performed using Lipofectamine 3000 (#L3000008, Invitrogen).

### Statistical Analysis

All data were shown as mean ± SD and analyzed on SPSS 22.0 software. All experiments were technically replicated three times. The unpaired Student's *t*‐test was used to analyze the differences between two groups. Fisher exact test was applied to analyze categorical data. Survival curves were constructed using the Kaplan–Meier method, and comparisons between graft survival times were analyzed with the Gehan–Breslow–Wilcoxon test. Linear correlations were evaluated using the Pearson correlation coefficient. Values of *p* < 0.05 were considered statistically significant.

## Conflict of Interest

The authors declare no conflict of interest.

## Author Contributions

B.N., C.Y., J.Z., and Z.H. contributed equally to this work. B.S., M.G., and Z.W. designed the research. B.N., C.Y., J.Z., and Z.H. performed most of the experiments. M.Z. performed in vivo experiments and RNA‐seq analyses. D.F., Q.S., and X.S. helped with molecular experiments. J. M. and L.S. contributed to the clinical sample collection. B.N. analyzed the data and wrote the manuscript. L.S., B.S., M.G., and Z.W. supervised the project and provided funding.

## Supporting information



Supporting Information

## Data Availability

The raw sequence data reported in this paper have been deposited in the Genome Sequence Archive in National Genomics Data Center, China National Center for Bioinformation / Beijing Institute of Genomics, Chinese Academy of Sciences (GSA: CRA021234) that are publicly accessible at https://ngdc.cncb.ac.cn/gsa.
