# Supplementary material for: Rictor Ameliorates Acute Antibody‐Mediated Rejection Following Kidney Transplantation by Suppressing Macrophage M1 Polarization Through p65‐NLRP3 Axis
Source: Adv Sci (Weinh). 2025 Jun 20;12(34):e17119. doi: 10.1002/advs.202417119 (PMC12442627; doi:10.1002/advs.202417119)
Supplement: Supplementary file 1 — Supporting Information [file ADVS-12-e17119-s001.docx]

Rictor ameliorates acute antibody-mediated rejection following kidney transplantation by suppressing macrophage M1 polarization through p65-NLRP3 axis

Bin Ni, Chengcheng Yang, Junqi Zhang, Zhou Hang, Ming Zheng, Dengyuan Feng, Qinghuan Shen, Jinxu Miao, Xulin Sun, Li Sun, Baixin Shen^*^, Min Gu^*^ and Zijie Wang^*^

Supplementary materials

Table S1: Primer sequences.

| Gene | Primer sequences (5’-3’) |
| --- | --- |
| Rictor | F: AGCTGGGCCATCTGAATAAC |
|  | R: GATGTCTTCATAATTAAAGCCCAGT |
| IL-1β | F: TTCCTGTTGTCTACACCAATGC |
|  | R: CGGGCTTTAAGTGAGTAGGAGA |
| TNFα | F: CCTCTCTAATCAGCCCTCTG |
|  | R: GAGGACCTGGGAGTAGATGAG |
| iNOS | F: GTT​CTC​AGC​CCA​ACA​ATA​CAA​GA |
|  | R: GTG​GAC​GGG​TCG​ATG​TCA​C |
| CD86 | F: TGTTTCCGTGGAGACGCAAG |
|  | R: TTGAGCCTTTGTAAATGGGCA |
| TGF-β1 | F: GGAGAGCCCTGGATACCAAC |
|  | R: CAACCCAGGTCCTTCCTAAA |
| CD206 | F: CTC​TGT​TCA​GCT​ATT​GGA​CGC |
|  | R: CGG​AAT​TTC​TGG​GAT​TCA​GCT​TC |
| Arg1 | F: CAGAAGAATGGAAGAGTCAG |
|  | R: CAGATATGCAGGGAGTCACC |
| IL-10 | F: GCTCTTACTGACTGGCATGAG |
|  | R: CGCAGCTCTAGGAGCATGTG |
| IL-6 | F: TAGTCCTTCCTACCCCAATTTCC |
|  | R: TTGGTCCTTAGCCACTCCTTC |
| NLRP3 | F: TGGATGGGTTTGCTGGGAT |
|  | R: CTGCGTGTAGCGACTGTTGAG |
| p65 | F: AGGCTTCTGGGCCTTATGTG |
|  | R: TGCTTCTCTCGCCAGGAATAC |
| PDLIM2 | F: TGGGGCTTCCGAATTAGCG |
|  | R: CGCGTGTAGCATGTTCTCTG |
| Trim21 | F: GGGAGGAGGTCACCTGTTCTA |
|  | R: GGGAGGAGGTCACCTGTTCTA |
| RNF182 | F: TACAATCGGTACAACCTGAAGC |
|  | R: GGCTGCTGACTTCGTCATCC |
| SOCS1 | F: CTGCGGCTTCTATTGGGGAC |
|  | R: AAAAGGCAGTCGAAGGTCTCG |
| Trim7 | F: ACAGAAACAGAATGAGAACCTGG |
|  | R: GCTCAGTGTGCTTTTGAACTCC |
| GAPDH | F: GAA​GGT​CGG​TGT​GAA​CGG​AT |
|  | R: CCCATTTGATGTTAGCGGGAT |


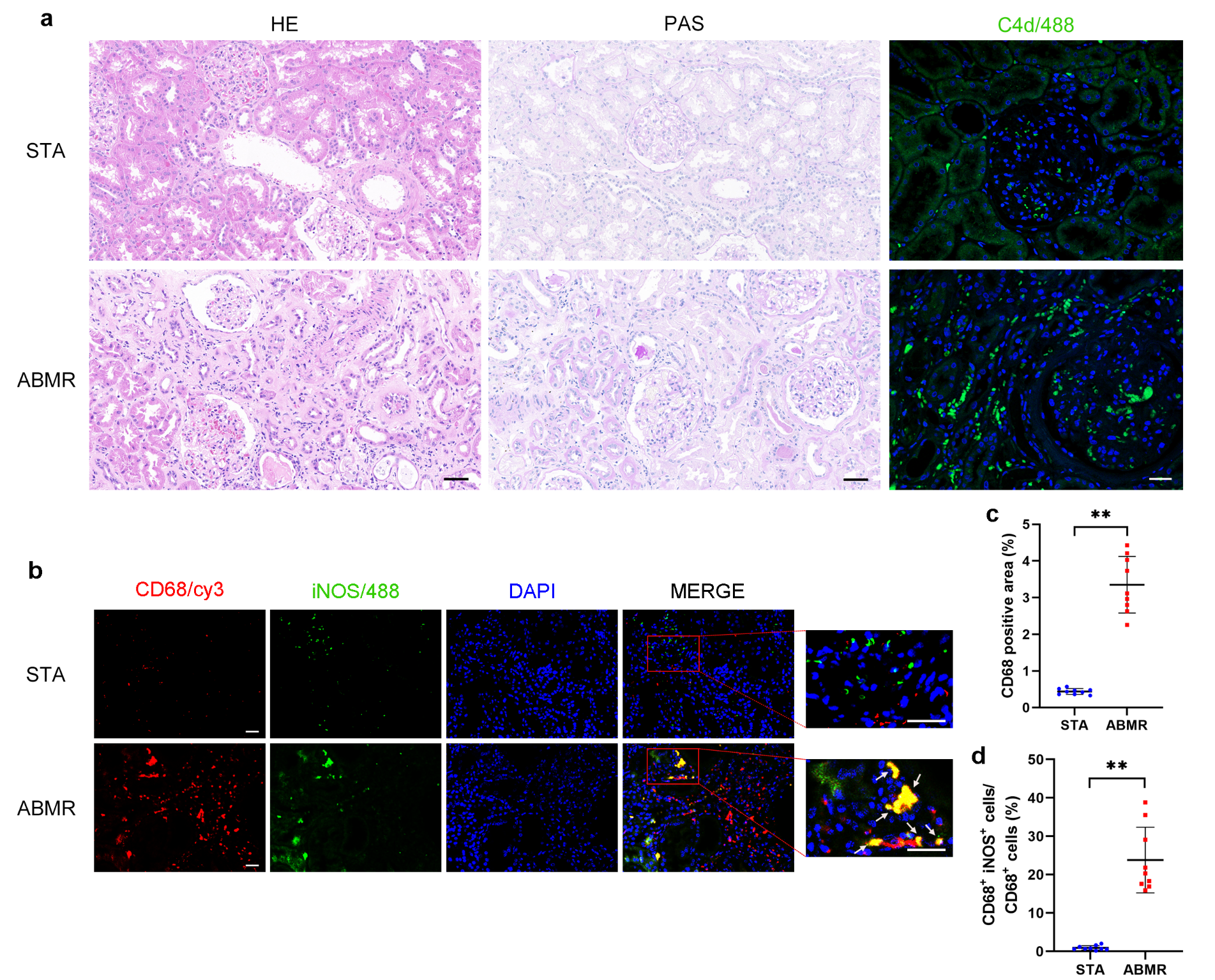


Figure S1: M1 macrophages in patients with stable (STA) and ABMR.

(a) Histologic images of kidney graft sections from patients with STA and ABMR stained with hematoxylin and eosin (H&E) and periodic acid Schiff (PAS). Original magnification ×200. Bar = 50 μm. Representative immunofluorescence (IF) staining for C4d in kidney grafts. Original magnification ×400. Bar = 20 μm. (b-d) Representative IF staining and quantitative analysis for CD68 and iNOS in kidney grafts. White arrows indicate co-staining-positive cells. Original magnification ×400, Bar = 20 μm, n = 9.


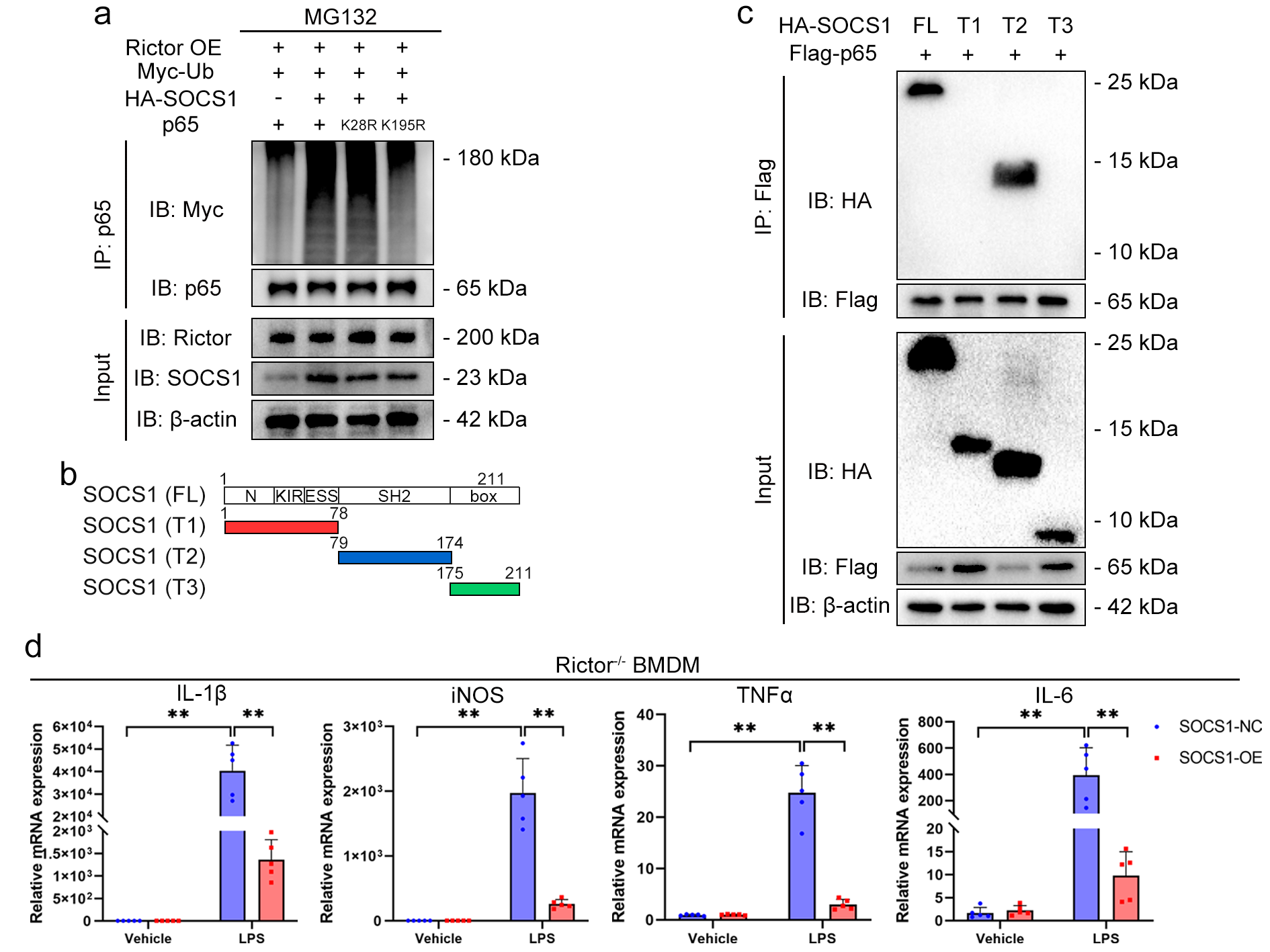


Figure S2: SOCS1 promotes p65 degradation at the K195 site through the SH2-domain.

a: Co-IP assay for the ubiquitination of p65 in Rictor OE HEK293T cells transfected with Myc-ubiquitin, HA-SOCS1 and different mutant p65 plasmids. b: Diagram of the different domains of the SOCS1 constructs and truncated plasmids. c: Co-IP assay for the interaction between p65 and different domains of SOCS1. d: qRT-PCR analysis showing the mRNA levels of *IL-1β*, *iNOS*, *TNFα* and *IL-6* in Rictor^-/-^ BMDMs among groups as indicated (n = 5). ** P* < 0.05; ** *P* < 0.01. Data are presented as mean ± SD.
